# Urachal cyst: a rare cause of an abdominal mass in a Ghanaian adult female: a case report

**DOI:** 10.1093/jscr/rjaf356

**Published:** 2025-05-31

**Authors:** Ishmael Kyei, Samuel Mensah, Joshua Tei Shiako, Emmanuel Owusu-Ansah, Christopher Aboah, Joseph Yorke

**Affiliations:** Department of Surgery, Kwame Nkrumah University of Science and Technology, Kumasi, Ghana; Department of Surgery, Kwame Nkrumah University of Science and Technology, Kumasi, Ghana; Department of Surgery, Komfo Anokye Teaching Hospital, Kumasi, Ghana; Department of Surgery, Komfo Anokye Teaching Hospital, Kumasi, Ghana; Department of Surgery, Komfo Anokye Teaching Hospital, Kumasi, Ghana; Department of Surgery, Kwame Nkrumah University of Science and Technology, Kumasi, Ghana

**Keywords:** urachus, urachal cyst, abdominal mass

## Abstract

Anomalies of the urachus, such as urachal cyst, albeit rare among the adult populace, pose an arsenal of myriad presentations among such age groups, leading to a high rate of misdiagnosis. Late diagnosis can predispose affected individuals to several complications such as cyst rupture, cyst infection with attending sepsis, fistula formation as well as neoplastic changes. Owing to this, a high index of suspicion is prudent for timely diagnosis. We present a case of a 48-year-old perimenopausal woman who presented to the general clinic with recurrent abdominal pain of 5 months duration with no associated symptoms. An abdominal computed tomography scan revealed a urachal cyst, which was managed surgically.

## Introduction

Urachal cysts, together with patent urachus, vesicourethral diverticulum and umbilical-urachal sinus, are the four common urachal anomalies associated with embryonic bladder formation [1–3]. Urachal cysts, the commonest, are mostly seen in children [3, 4], and rarely present in adulthood [5, 6]. In view of that, its incidence among the adult population is not well established [1]. It usually arises from an incomplete obliteration of the urachus, a fibromuscular stalk which connects the umbilical cord to the developing bladder during foetal development [7–9]. and serves as a conduit for drainage [10]. This results in a retained cyst along the median umbilical ligament in post-natal life [5]. It has an incidence rate of 1/5000 live births, and it is mostly asymptomatic [4, 5, 10]. However, when infected it presents symptoms that mimic pelvic inflammatory disease and other intra-abdominal disease processes [1, 11]. We report a case of a 48-year-old perimenopausal woman presenting with a 5-month history of abdominal pain, an abdominal computed tomography (CT) confirmed a urachal cyst, and she was managed surgically.

## Case presentation

A 48-year-old perimenopausal woman presented to the clinic with a 5-month history of abdominal pain. The pain was central, non-colicky and dull. The pain was constant with occasional exacerbation likely from the pressure effect of the mass. She had no history of chronic diseases.

**Figure 1:**
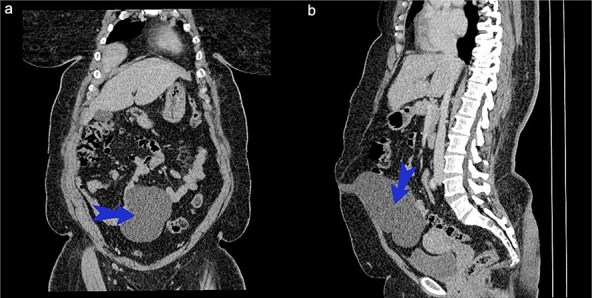
(a) CT scan of abdomen coronary view. (b) CT scan of abdomen sagittal view.

On examination, there was a well-defined lower abdominal mass measuring ~10 × 7 cm with a smooth surface. It was soft, fluctuant and mobile in the horizontal plane. No organomegaly observed. Lab investigations were all within normal limits. An abdominal CT scan (Fig. 1a and b) showed a cystic well-defined lesion posterior to the anterior abdominal wall from the level of the umbilicus to the dome of the bladder.

**Figure 2:**
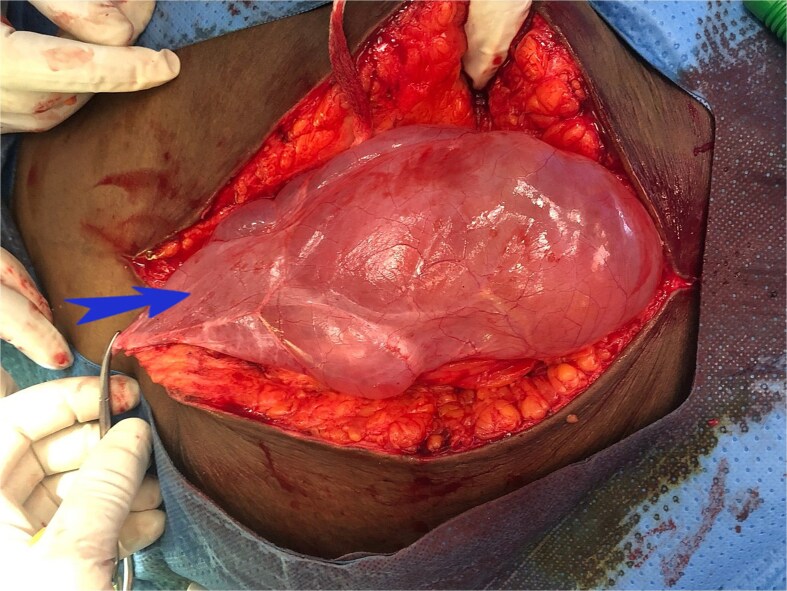
Intraoperative image showing urachal cyst.

**Figure 3:**
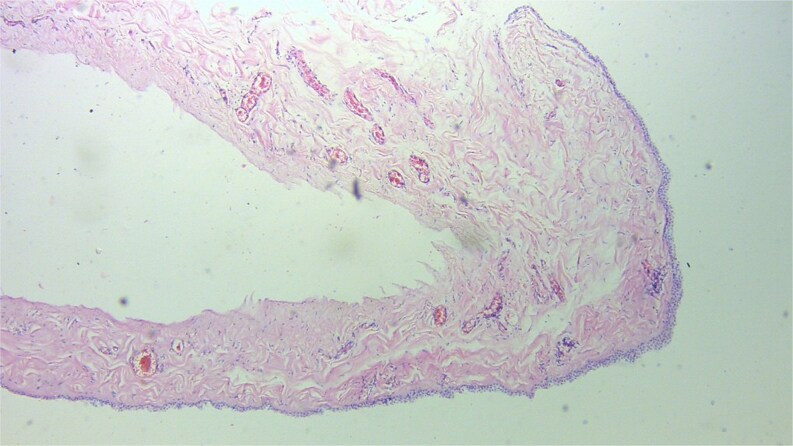
Photomicrograph of cyst ×10 magnification.

At surgery a cystic lesion was found attached the anterior abdominal wall at the umbilicus, posterior to the fascia (Fig. 2). Blunt and sharp dissection were used to separate the cyst from the anterior abdominal wall towards the bladder, excision of the urachal cyst was done, and the contiguous duct to the bladder excised with a disc of bladder wall tissue. The bladder defect was repaired the abdomen was closed. The bladder was drained continuously for 10 days. Histology confirmed a urachal cyst (Fig. 3).

## Discussion

Embryonically, the urachus is a remnant of the primitive bladder dome [4, 9]. It is a derivative of the allantois and is a fibromuscular cord that emanates from the anterior aspect of the bladder wall and extends cranially to the umbilicus [1, 5, 10]. Urachal anomalies stem from failure of the urachus to regress completely during foetal development [12, 13]. Persistence of part of it or its entirety in postnatal life may culminate in 1 of 4 urachal anomalies [1, 14].

Regarding urachal anomalies, urachal cyst is reported to be the most common type, and it ensues when both proximal and distal ends of the fibrous urachus obliterate creating a cystic cavity in the mid-portion [3–5]. It is rarely seen in adults hence its incidence among such age group is unknown [1, 4, 9, 10]. Urachal cysts are usually asymptomatic, warranting intervention when complicated [4]. Infected urachal cyst predisposes affected individuals to a number of clinical complications such as cyst rupture, stone formation, formation of bladder fistula, peritonitis, sepsis as well as neoplastic changes [1, 15].

The patient’s age and the chronicity of the abdominal pain without significant associations, posed a clinical challenge delaying diagnosis. A high index of suspicion is needed for diagnosis among the adult population as the presentation may mimic other clinical presentations like pelvic inflammatory disease, urinary tract infection, Merkel’s diverticulitis and bladder carcinoma [4].

CT or magnetic resonance imaging (MRI) is the most sensitive imaging modality for diagnosing urachal anomalies [1, 4]. These modalities assess the spatial relationship between these anomalies and surrounding structures [1], necessary in planning the required surgical approach [3]. However, in Ghana and low-and middle income countries, MRI or CT scan services are not widely available and hence, ultrasound tends to be the most convenient imaging modality for diagnosing most intra-abdominal pathologies. The unusual presentation in our index case influenced the use of CT scan. Studies by Allen *et al.* [3] reported that ultrasound is effective in diagnosing urachal cysts.

Although the approach to the management of urachal cyst is not well standardized, the main stay of treatment that is deemed curative is surgical excision of the embryonic remnants [1]. Traditionally, a two-stage approach where there is prior incision and drainage of the cyst followed by excision of the cyst was used in the management of complicated urachal cyst [1]. The single-stage method was associated with more complications such as wound infection and urine leak as well as protracted hospital stay compared to the two-stage approach [2]. These complications tend to be lower if prophylactic antibiotics are given.

Our patient had excision of urachal cyst done she received 2 g of ceftriaxone at induction prophylactically with no postoperative complications. Although laparoscopic surgery is the standard for urachal cyst excision currently our centre’s laparoscopic expertise is limited to diagnostic and surgeries like cholecystectomy and appendectomy**.** Her four days stay in the hospital was shorter than the average hospital stay for the two-stage approach reported in literature.

## Conclusion

Urachal cysts are infrequent in adults and pose a clinical challenge owing to their vague presentation. Single stage excision under antibiotic cover is a viable treatment option in uncomplicated cases.

## References

[1] Elkbuli A, Kinslow K, Ehrhardt JD Jr, et al. Surgical management for an infected urachal cyst in an adult: case report and literature review. Int J Surg Case Rep 2019;57:130–3. 10.1016/j.ijscr.2019.03.041 30959359 PMC6453943

[2] Minevich E, Wacksman J, Lewis AG, et al. The infected urachal cyst: primary excision versus a staged approach. J Urol 1997;157:1869–72. 10.1016/S0022-5347(01)64889-4 9112551

[3] Allen JW, Song J, Velcek FT. Acute presentation of infected urachal cysts: case report and review of diagnosis and therapeutic interventions. Pediatr Emerg Care 2004;20:108–11. 10.1097/01.pec.0000113880.10140.19 14758308

[4] Jayakumar S, Darlington D. Acute presentation of urachal cyst: a case report. Cureus 2020;12:e8220. 10.7759/cureus.8220 PMC7306636 32582481

[5] Goldman IL, Caldamone AA, Gauderer M, et al. Infected urachal cysts: a review of 10 cases. J Urol 1988;140:375–8. 10.1016/S0022-5347(17)41612-0 3398141

[6] Faye PM, Gueye ML, Thiam O, et al. Infected urachal cyst in an adult, report of two observations. Int J Surg Case Rep 2022;97:107394. 10.1016/j.ijscr.2022.107394 35834928 PMC9403061

[7] Begg RC . The urachus: its anatomy, histology and development. J Anat 1930;64:170–83. https://www.ncbi.nlm.nih.gov/pmc/articles/PMC1250190/17104266 PMC1250190

[8] Binnie JF . Development of the urachus. JAMA 1906;XLVII:109–10. 10.1001/jama.1906.25210020029002h

[9] Hammond G, Yglesias L, Davis JE. The urachus, its anatomy and associated fasciae. Anat Rec 1941;80:271–87. 10.1002/ar.1090800302

[10] Moore KL, Persaud TVN, Torchia MG. The Developing Human E-Book [Internet]. Philadelphia PA: Elsevier Health Sciences, 2011.

[11] Spataro RF, Davis RS, McLachlan MS, et al. Urachal abnormalities in the adult. Radiology 1983;149:659–63. 10.1148/radiology.149.3.6647841 6647841

[12] Choi YJ, Kim JM, Ahn SY, et al. Urachal anomalies in children: a single center experience. Yonsei Med J 2006;47:782–6. 10.3349/ymj.2006.47.6.782 17191305 PMC2687816

[13] Wilson AL, Gandhi J, Seyam O, et al. Urachal anomalies: a review of pathological conditions, diagnosis, and management. Transl Res Anat 2019;16:100041. 10.1016/j.tria.2019.100041

[14] Muniraman H, Sardesai T, Sardesai S. Disorders of the umbilical cord. Pediatr Rev 2018;39:332–41. 10.1542/pir.2017-0202 29967078

[15] Masuko T, Nakayama H, Aoki N, et al. Staged approach to the urachal cyst with infected omphalitis. Int Surg 2006;91:52–6. 16706104

